# Quantum Secure Multi-Party Summation Using Single Photons

**DOI:** 10.3390/e25040590

**Published:** 2023-03-30

**Authors:** Wan-Qing Wu, Ming-Zhe Xie

**Affiliations:** 1 School of Cyber Security and Computers, Hebei University, Baoding 071002, China; 2 Key Laboratory on High Trusted Information System in Hebei Province, Hebei University, Baoding 071002, China

**Keywords:** quantum cryptography, quantum secure multi-party summation, quantum computing

## Abstract

In this paper, we propose a secure multi-party summation based on single photons. With the help of a semi-honest third party, *n* participants can simultaneously obtain the summation result without revealing their secret inputs. Our protocol uses single photon states as the information carriers. In addition, each participant with secret input only performs simple single-particle operators rather than particle preparation and any complex quantum measurements. These features make our protocol more feasible to implement. We demonstrate the correctness and security of the proposed protocol, which is resistant to participant attack and outside attack. In the end, we compare in detail the performance of the quantum summation protocol in this paper with other schemes in terms of different indicators. By comparison, our protocol is efficient and easy to implement.

## 1. Introduction

As a fundamental primitive in modern cryptography, secure multi-party computation (SMC) enables *n* (*n* ≥ 2) parties to jointly compute a function based on their private inputs while keeping these inputs private. Yao [1] first put forward the idea of SMC in 1982. SMC has a wide range of applications in secret ballot elections [2], private bidding and auctions [3,4], secret sharing [5], data mining [6], and so on. However, the appearance of Shor’s algorithm [7] and Grover’s algorithm [8] threatened the security of classical cryptography protocols based on difficult mathematical problems. To cope with this problem, people considered using the principles of quantum mechanics in cryptography protocols, which led to the birth of various interesting research fields, such as quantum key distribution (QKD) [9,10,11,12], quantum secret sharing (QSS) [13,14] and, the area of research in which this article is based, secure multi-party quantum computation (SMQC). Of these, the QKD field has made many notable advances. In 2018, Lucamarini et al. [9] presented overcoming the rate–distance limit of quantum key distribution protocol without quantum repeaters. Lin et al. [10] proposed a simple security analysis of the phase-matching measurement-device-independent quantum key distribution protocol. In 2022, Gu et al. [11] proposed an experimental measurement-device-independent-type quantum key distribution protocol with flawed and correlated sources. Xie et al. [12] presented a breaking the rate-loss bound of quantum key distribution protocol with asynchronous two-photon interference.

Secure multi-party quantum summation (SMQS) is a subfield of secure multi-party quantum computing and has gained much attention these years. It can enable *n* participants to jointly calculate a summation without revealing any participant’s secret to others. Quantum summation can be applied to a variety of fields, such as quantum voting [15,16,17,18], quantum anonymous ranking [19,20], and quantum private equality comparison [21,22,23]. Designing efficient and practical quantum summation protocols is thus significant.

To date, a variety of quantum summation protocols have been proposed by using various quantum resources. For example, in 2006, Hillery et al. [24] proposed a multi-party summation protocol with the two-particle N-level entangled states. In 2010, Chen et al. [25] presented a binary quantum summation protocol based on GHZ entangled states. In 2016, Shi et al. [26] proposed an interesting quantum algorithm to calculate multi-party summation and multiplication. The calculation result is safely translated into the corresponding phase information by using the quantum Fourier transform. In 2017, Zhang et al. [27] devised a multi-party quantum summation protocol that requires three-particle entangled states to be shared among users. Liu et al. [28] presented a quantum summation protocol by sharing multi-particle entangled states, including bell state, among users. Since their protocol’s quantum communication is two-way, it is vulnerable to Trojan horse attack. In 2019, Ji et al. [29] designed two quantum summation protocols by employing entanglement swapping property between the *d*-level Bell state and the *d*-level *n*-partite cat state. In 2021, Wu et al. [30] proposed a multi-party quantum summation protocol based on the *d*-level Bell states. In 2022, Hayashi et al. [31] utilized phase GHZ states to construct a secure quantum modulo summation protocol that had the advantage of verifiability based on self-testing, allowing it to perform in worse security environments.

In the above proposed QSMS protocols, most protocols depend on sharing a multi-particle entangled state among users. Nevertheless, these protocols encounter a problem in practical application, that is, it is difficult to prepare the information carriers (multi-particle entangled states) with current technology. With this in mind, some papers designed the QSMS protocol in single particles. In 2014, Zhang et al. [32] employed single photons in both polarization and spatial-mode degrees of freedom to design a high-capacity quantum summation protocol. However, their protocol has a security vulnerability. A malicious participant can use an intercept-resend attack to obtain the next participant’s secret [33]. In 2019, Zhang et al. [34] used a set of mutually unbiased bases in a single *d*-level quantum system to construct a multi-party quantum summation protocol. Unlike other protocols that sum a whole string of numbers, their protocol can only sum a single number. In 2020, Duan et al. [35] used single photons to construct a quantum summation protocol for transmission in a circular way. In their protocol, randomly selected encoded particles need to be measured to check the security of the communication, which prevents the final result from being calculated as each participant wishes, thus greatly limiting the practical application of their protocol.

Based on the above, publishing safe, efficient and easy to implement protocols is necessary. So, we propose a novel quantum secure multi-party summation protocol using single photons. The secret inputs are encoded as single photons and then encrypted with a simple unitary operation. Relying on this method, our protocol can achieve efficient and easy-to-implement goals with fewer quantum resources. The rest of this paper is organized as follows. In Section 2, we propose a three-party quantum summation protocol and discuss the security of the presented protocol. In Section 3, we generalize the proposed three-party quantum summation protocol to multi-part and analyze the security of the multi-party protocol. In Section 4, we compare the previous quantum summation protocols with our multi-party quantum summation protocol. Finally, we make a conclusion in Section 5.

## 2. The Three-Party Quantum Summation Protocol

### 2.1. Proposed Protocol

Secure three modulo-2 summation is defined as follows. Suppose that there are three participants named Alice, Bob and Charlie, who all own the same length of secret input $x_{A}$, $x_{B}$, $x_{C}$, respectively, where $x_{A}=\left(x_{A1},x_{A2},\cdots ,x_{Am}\right),x_{B}=\left(x_{B1},x_{B2},\cdots ,x_{Bm}\right)$ and $x_{C}=\left(x_{C1},x_{C2},\cdots ,x_{Cm}\right)$. Here, $x_{At},x_{Bt},x_{Ct}\in \left\{0,1\right\}$ for t=1,2,⋯,m. They calculate the sum by encoding the information on the information carrier, that is, $(x_{A}+x_{B}+x_{C})mod2$. (Note that $\left(x_{A}+x_{B}+x_{C}\right)mod2=x_{A1}⊕x_{B1}⊕x_{C1}$, $x_{A2}⊕x_{B2}⊕x_{C2},\cdots ,x_{Am}⊕x_{Bm}⊕x_{Cm}$. Here, “⊕” denotes the addition modulo 2).

In addition, it should satisfy the following requirements (please refer to [36]):

**Correctness:** The result of modulo-2 summation of all participants’ secret inputs should be correct.

**Fairness:** All participants receive the summation result simultaneously.

**Privacy:** Participants’ secret inputs are private. In other words, no participant can learn about other participants’ secret inputs, even though the participant can launch various quantum attacks and up to n-2 participants are allowed to conspire but not with TP and an outside eavesdropper (here, *n* is the number of participants in the protocol).

**Security:** An outside eavesdropper cannot learn any information about each participant’s secret input without being detected.

In the following, we propose a secure protocol to accomplish this task with the help of a third party (TP). The TP is assumed to be semi-honest but non-collusive, that is to say, TP is allowed to launch various attacks by using different quantum resources under the premise of loyally execution of the protocol, but he cannot collude with other participants. The classical and quantum channel used in our protocol are assumed to be authenticated and noiseless, respectively.

All participants agree on the following forms:

(1) $$\begin{array}{cc} \; & {|0\rangle }_{0}={|0\rangle ,|0\rangle }_{1}=|+\rangle ,\\ \; & {|1\rangle }_{0}={|1\rangle ,|1\rangle }_{1}=|-\rangle . \end{array}$$

**Figure 1:**
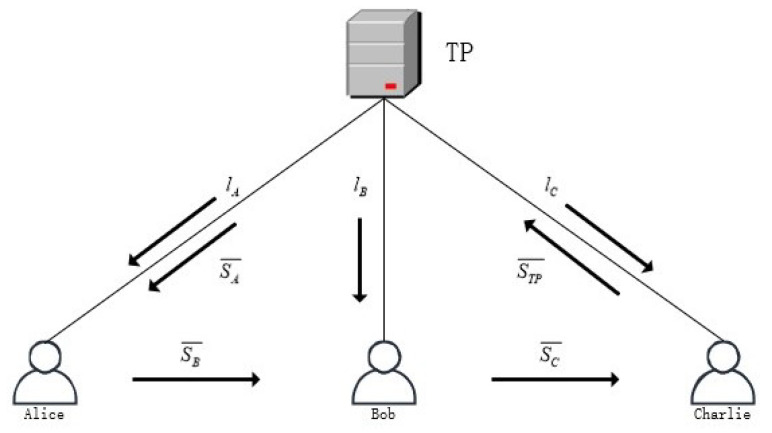
The process of three-party quantum summation.

Here, we denote the two orthogonal states of a qubit as ${|0\rangle }_{0}$ and ${|1\rangle }_{0}$, respectively, and define ${|0\rangle }_{1}=|+\rangle$, ${|1\rangle }_{1}=|-\rangle$, where $|+\rangle =\frac{1}{\sqrt{2}}(|0\rangle +|1\rangle ),|-\rangle =\frac{1}{\sqrt{2}}(|0\rangle -\left|1\rangle \right)$. In addition, ${|0\rangle }_{0}=|0\rangle$, ${|1\rangle }_{0}=|1\rangle$ represent the classical bits 0, 1, respectively. The specific steps of three-party protocol are shown in Figure 1.

Our three-party protocol works as follow:

**Step 1:** TP sends a secret key sequence $l_{A}$($l_{B}$, $l_{C}$) to Alice (Bob, Charlie) via the quantum key distribution protocol [11] beforehand, where $l_{A}=\left(l_{A1},\ldots ,l_{Am}\right)(l_{B}=\left(l_{B1},\ldots ,l_{Bm}\right),$$l_{C}=\left(l_{C1},\ldots ,l_{Cm}\right)),l_{At}\left(l_{Bt},l_{Ct}\right)\in \left\{0,1\right\},t=1,2,\cdots ,m.$

**Step 2:** According to the secret key sequence $l_{A}$ previously shared with Alice, TP generates *m* copies of single photon states and uses these particles to construct a sequence

$$S_{A}=\left\{{|0\rangle }_{l_{A1}}{,|0\rangle }_{l_{A2}},\ldots ,{|0\rangle }_{l_{Am}}\right\}.$$

To ensure the security of particle transmission, TP prepares *m* decoy photons, which randomly in $\left\{|0\rangle ,|1\rangle ,|+\rangle ,|-\rangle \right\}$. Then, he inserts the decoy photons into $S_{A}$ at random positions and records the insertion positions. Denote the new sequence by $\bar{S_{A}}$. Finally, TP sends $\bar{S_{A}}$ to Alice.

**Step 3:** After confirming that Alice has received all the particles from TP, Alice checks the security of the transmission of $\bar{S_{A}}$ with TP. Specifically, TP announces the insertion positions and the bases of the decoy photons in $\bar{S_{A}}$ to Alice. Then, based on the announced information, Alice measures these decoy states on the correct bases and publishes the measurement results to TP. Subsequently, according to Alice’s measurements, TP checks for the presence of eavesdroppers in the quantum channel. If the error rate is higher than the threshold determined by the channel noise, TP cancels this protocol and restarts it. Otherwise, TP proceeds to the next steps.

**Step 4:** After determining that the transmission has not eavesdropped, Alice obtains $S_{A}$ by extracting decoy photons from $\bar{S_{A}}$. Then, Alice encodes his secret input $x_{A}$ on the sequence $S_{A}$. Concretely, Alice performs the unitary operation $U_{Y}^{x_{At}}$ on the *t* th particle of $S_{A}$. Here, the operators are defined by [37],

(2) $$U_{Y}^{1}=iY=\left[\begin{array}{cc} 0 & 1\\ -1 & 0 \end{array}\right],$$

(3) $$U_{Y}^{0}=I=\left[\begin{array}{cc} 1 & 0\\ 0 & 1 \end{array}\right].$$

Obviously, after Alice finishes the encoding operations, the quantum states in $S_{A}$ are changed into $\left\{|x_{A1}\rangle_{l_{A1}},|x_{A2}\rangle_{l_{A2}},\ldots ,{|x_{Am}\rangle }_{l_{Am}}\right\}$. The transformed sequence is denoted as $S_{B}$. Alice prepares *m* decoy states that are randomly in $\left\{|0\rangle ,|1\rangle ,|+\rangle ,|-\rangle \right\}$ and inserts them in $S_{B}$ to form a new sequence $\bar{S_{B}}$. Afterward, Alice sends $\bar{S_{B}}$ to Bob. After receiving the sequence $\bar{S_{B}}$, Bob performs the same operation as Alice, namely, security checking and encoding secret information. In addition, Bob performs the Hadamard operation $H^{l_{Bt}}$ on the *t* th particle of $S_{B}$ according to the received secret key sequence $l_{B}$:

(4) $$H^{1}=H=-\frac{1}{\sqrt{2}}\left[\begin{array}{cc} 1 & 1\\ 1 & -1 \end{array}\right],$$

(5) $$\begin{array}{c} H^{0}=I=\left[\begin{array}{cc} 1 & 0\\ 0 & 1 \end{array}\right] \end{array}.$$

After completing the above operations, Bob obtains a sequence $S_{C}$. Then, he randomly places decoy photons into the sequence $S_{C}$ to form a new sequence $\bar{S_{C}}$. Finally, Bob sends $\bar{S_{C}}$ to Charlie. When Charlie receives the sequence $\bar{S_{C}}$, he performs the same operations as Bob, namely security checking, encoding the secret information and the secret key. Then Charlie sends the resulting new sequence $\bar{S_{TP}}$ to TP.

**Step 5:** After receiving the sequence $\bar{S_{TP}}$, TP and Charlie jointly check the security of the transmission channel. TP obtains the sequence $S_{TP}$ by extracting decoy photons after confirming that the channel is safe. Then, TP computing $L_{1}=l_{A1}⊕l_{B1}⊕l_{C1},$$L_{2}=l_{A2}⊕l_{B2}⊕l_{C2},\ldots ,L_{m}=l_{Am}⊕l_{Bm}⊕l_{Cm}$. If $L_{t}=1\left(t=1,2,\ldots ,m\right)$, TP will perform the Hadamard operation on the *t* th particle of $S_{TP}$. Otherwise, the particles in $S_{TP}$ will remain the same. After the above operation, TP measures the particles with the Z basis. Then, TP can acquire the summation $x_{A1}⊕x_{B1}⊕x_{C1},x_{A2}⊕x_{B2}⊕x_{C2},\ldots ,x_{Am}⊕x_{Bm}⊕x_{Cm}$. Finally, TP announces the summation result to Alice, Bob, and Charlie via a public channel.

To illustrate our protocol more clearly, we will take an example with three participants, Alice, Bob and Charlie. For the sake of convenience, we will omit the security checking.

**Sample 1:** We assume the secret input of Alice Bob and Charlie are $x_{A}$ = (010), $x_{B}$ = (011) and $x_{C}$ = (011), respectively.

First, TP sends secret key sequences to Alice, Bob and Charlie, where the secret key sequence is $l_{A}$ = (001), $l_{B}$ = (010) and $l_{C}$ = (011), respectively. Then, TP generates the three copies of single photon states ${|0\rangle }_{0}{,|0\rangle }_{0},{|0\rangle }_{1}$ and sends them to Alice. Alice applies the encoding operations on the signal particles according to the secret numbers $x_{A}$ and transmits the encoded particles to Bob. Subsequently, Bob encodes the received particles according to his secret input $x_{B}$ and secret key sequence $l_{B}$ and transmits them to Charlie. When Charlie receives the signal particles, he performs the same operation as Bob. He encodes the particles according to his secret input $x_{C}$ and secret key sequence $l_{C}$ and then transmits them to the TP. The corresponding operations and the changes in quantum states are shown in Table 1.

After the above steps, TP calculates the sum of the keys $L_{1}$ = 0, $L_{2}$ = 0, $L_{3}$ = 0. He performs the operation I⊗I⊗I based on the calculation result and yields the states |0〉,|1〉,|0〉. Finally, TP measures these particles in the Z basis and obtains the summation $x_{A}⊕x_{B}⊕x_{C}$ = 010.

**Channel loss of the cited QKD protocol:** Although we assumed that the quantum channel is noiseless, channel loss is a major issue in the construction of QKD, so it is discussed here. The QKD protocol [11] used in our protocol utilizes coherent states to against realistic flawed sources and ensure security by adopting the reference technique. A proof-of-principle experiment in Ref. [11] demonstrates the feasibility of the QKD protocol in terms of resistance to channel loss.

### 2.2. Correctness

In this part, we discuss the correctness of the proposed three-party quantum summation protocol. Here, we show that all participants provide their secret inputs honestly, and they can eventually obtain the correct summation result:

(6) $$x_{A1}+x_{B1}+x_{C1}\left(mod2\right),x_{A2}+x_{B2}+x_{C2}\left(mod2\right),\ldots ,x_{Am}+x_{Bm}+x_{Cm}\left(mod2\right)$$

By deriving Equations (2)–(5), we can obtain the following equations:

(7) $$\begin{array}{c} H^{2}=U_{Y}^{2}=I \end{array}$$

(8) $$\begin{array}{c} HU_{Y}H=-U_{Y} \end{array}$$

(9) $$\begin{array}{c} HU_{Y}=-U_{Y}H \end{array}$$

Before the protocol is implemented, Alice, Bob and Charlie negotiate a coding rule (1), where |0〉,|1〉 represent the classical bits 0, 1, respectively. Without loss of generality, we consider only the operation performed on the j-th particle. Suppose that the initial state of the *t*-th particle is ${|S\rangle }_{j}\epsilon \left\{|0\rangle ,|+\rangle \right\}$. In step 4, Alice, Bob and Charlie perform their unitary operations on ${|S\rangle }_{t}$, and the state of the *t*-th particle will change to the following form:

(10) $$|S^{*}\rangle_{t}=H^{l_{Ct}}U_{Y}^{x_{Ct}}H^{l_{Bt}}U_{Y}^{x_{Bt}}U_{Y}^{x_{At}}{|S\rangle }_{t}$$

Obviously, we know

(11) $$\begin{array}{c} \begin{array}{cc} \; & U_{Y}{|0\rangle }_{0}=U_{Y}|0\rangle =-|1\rangle ;\\ \; & U_{Y}{|0\rangle }_{1}=U_{Y}|1\rangle =|0\rangle ;\\ \; & U_{Y}{|1\rangle }_{0}=U_{Y}|+\rangle =|-\rangle ;\\ \; & U_{Y}{|1\rangle }_{1}=U_{Y}|-\rangle =-|+\rangle . \end{array} \end{array}$$

So, we can obtain the

(12) $$\begin{array}{c} \begin{array}{cc} \; & U^{1}{|0\rangle }_{0}={|0⊕1\rangle }_{0},\;U^{1}{|0\rangle }_{1}={|0⊕1\rangle }_{1},\;U^{1}{|1\rangle }_{0}={|1⊕1\rangle }_{0},\;U^{1}{|1\rangle }_{1}={|1⊕1\rangle }_{1},\\ \; & U^{0}{|0\rangle }_{0}={|0⊕0\rangle }_{0},\;U^{0}{|0\rangle }_{1}={|0⊕0\rangle }_{1},\;U^{0}{|1\rangle }_{0}={|0⊕1\rangle }_{0},\;U^{0}{|1\rangle }_{1}={|0⊕1\rangle }_{1}. \end{array} \end{array}$$

In addition, the Hadamard operator is equivalent to the interchange of Z basis (|0〉,|1〉) and X basis (|+〉,|−〉). This is expressed in the following forms:

(13) $${H|0\rangle }_{0}={|0\rangle }_{0⊕1}{,H|0\rangle }_{1}={|0\rangle }_{1⊕1}{,H|1\rangle }_{0}={|1\rangle }_{0⊕1}{,H|1\rangle }_{1}={|1\rangle }_{1⊕1}.$$

By Equations (12) and (13), $|S^{*}\rangle_{t}$ can be expressed in the following form:

(14) $$|S^{*}\rangle_{t}={|x_{At}⊕x_{Bt}⊕x_{Ct}\rangle }_{l_{At}⊕l_{Bt}⊕l_{Ct}}.$$

In step 5, TP performs unitary operations on $|S^{*}\rangle_{t}$ based on the sum of the secret keys. Through the above equations, we can derive that

(15) $$\begin{array}{c} \begin{array}{cc} H^{L_{t}}{|S^{*}\rangle }_{j} & =H^{L_{t}}H^{l_{Ct}}U_{Y}^{x_{Ct}}H^{l_{Bt}}U_{Y}^{x_{Bt}}U_{Y}^{x_{At}}{|S\rangle }_{t}\\ \; & =H^{L_{t}⊕l_{At}⊕l_{Bt}⊕l_{Ct}}U^{x_{At}⊕x_{Bt}⊕x_{Ct}}{|S\rangle }_{t}\\ \; & =|x_{At}⊕x_{Bt}⊕x_{Ct}\rangle_{L_{t}⊕l_{At}⊕l_{Bt}⊕l_{Ct}} \end{array} \end{array}$$

Clearly, $L_{t}⊕l_{At}⊕l_{Bt}⊕l_{Ct}$ = 0. So, we can further acquire

(16) $$H^{L_{t}}|S^{*}\rangle_{t}={|x_{At}⊕x_{Bt}⊕x_{Ct}\rangle }_{0}.$$

After TP performs Z basis measurement on the *t*-th particle of sequence $S_{TP}$, the particle collapse into classical information $x_{At}⊕x_{Bt}⊕x_{Ct}$. Therefore, when TP has finished performing Z basis measurements, the particles in sequence $S_{TP}$ will collapse into the sum of Alice, Bob and Charlie’s secret inputs.

It can be concluded that the output of the three-party quantum summation protocol is correct.

### 2.3. Security Analysis

This part will prove that the proposed three-party quantum summation protocol is secure against two kinds of threats: outside attack and participant attack. In the aspect of defending against the outside attack, we will show that an outside eavesdropper cannot learn any participant’s secret. In the aspect of the participant attack, we will show that the protocol is information-theoretically secure [38], that is, anyone including TP cannot obtain any information about other participants.

#### 2.3.1. Outside Attack

Obviously, the same protection measures are used for each transmission of the particles. Without loss of generality, here, we only analyze the security of the transmission of $\bar{S_{C}}$ against an outside eavesdropper, namely Eve. She strives to steal the secret inputs of participants. Hence, she could exploit any possible attack strategies but not collusion, such as the Trojan horse attack, the entanglement attack, the intercept–resend attack, the measure–resend attack. We will explain that our protocol is resistant to these attacks, and the specific analysis is as follows:

(**1**) **The Trojan horse attacks**

The Trojan horse attacks consist mainly of the delay-photon Trojan horse attack [39] and the invisible photon eavesdropping attack [40]. Since the particles of $\bar{S_{C}}$ are transmitting one-way, this protocol is naturally protected against the Trojan horse attacks from Eve.

(**2**) **The entangle-measure attack**

Eve cannot discover the difference between target and decoy photons. Therefore, she usually extracts some useful information by entangling her auxiliary particle |ε〉 with the one in $\bar{S_{C}}$ through a unitary operation $U_{E}$. Her behavior can be expressed as Equations (17) and (18):

(17) $$U_{E}|0\rangle |\varepsilon \rangle =a_{1}\left|0\rangle \right|\varepsilon_{00}\rangle +b_{1}\left|1\rangle \right|\varepsilon_{01}\rangle ,$$

(18) $$U_{E}|1\rangle |\varepsilon \rangle =a_{2}\left|0\rangle \right|\varepsilon_{10}\rangle +b_{2}\left|1\rangle \right|\varepsilon_{11}\rangle ,$$

where ${|a_{i}|}^{2}+{|b_{i}|}^{2}=1$ (*i* = 1, 2). If Eve’s operation does not introduce an error in the eavesdropping check, the following requirements are met:

(19) $$\begin{array}{c} \begin{array}{cc} \; & U_{E}|0\rangle |\varepsilon \rangle =\left|0\rangle \right|\varepsilon_{0}\rangle ,\\ \; & U_{E}|1\rangle |\varepsilon \rangle =\left|1\rangle \right|\varepsilon_{1}\rangle ,\\ \; & U_{E}|+\rangle |\varepsilon \rangle =\left|+\rangle \right|\varepsilon_{+}\rangle ,\\ \; & U_{E}|-\rangle |\varepsilon \rangle =\left|-\rangle \right|\varepsilon_{-}\rangle . \end{array} \end{array}$$

Taking $U_{E}|-\rangle |\varepsilon \rangle =\left|-\rangle \right|\varepsilon_{-}\rangle$ as an example, we expand both sides of the equation as follows:

(20) $$\begin{array}{c} \begin{array}{cc} \; & U_{E}|-\rangle |\varepsilon \rangle =\left|-\rangle \right|\varepsilon_{-}\rangle ,\\ ⟹ & \frac{1}{\sqrt{2}}(U_{E}|0\rangle |\varepsilon \rangle -U_{E}\left|1\rangle |\varepsilon \rangle \right)=\frac{1}{\sqrt{2}}(|0\rangle |\varepsilon_{-}\rangle -|1\rangle |\varepsilon_{-}\rangle ),\\ ⟹ & \frac{1}{\sqrt{2}}(a_{1}|0\rangle |\varepsilon_{00}\rangle +b_{1}\left|1\rangle \right|\varepsilon_{01}-a_{2}\left|0\rangle \right|\varepsilon_{10}-b_{2}\left|1\rangle \right|\varepsilon_{11}\rangle )=\frac{1}{\sqrt{2}}(|0\rangle |\varepsilon_{-}\rangle -|1\rangle |\varepsilon_{-}\rangle ),\\ ⟹ & \frac{1}{\sqrt{2}}[|0\rangle (a_{1}|\varepsilon_{00}\rangle -a_{2}|\varepsilon_{10}\rangle -|\varepsilon_{-}\rangle )+|1\rangle (b_{1}|\varepsilon_{10}\rangle -b_{2}|\varepsilon_{11}\rangle +|\varepsilon_{-}\rangle )]=0,\\ ⟹ & a_{1}|\varepsilon_{00}\rangle -a_{2}|\varepsilon_{10}\rangle -|\varepsilon_{-}\rangle =0,\\ \; & b_{1}|\varepsilon_{10}\rangle -b_{2}|\varepsilon_{11}\rangle +|\varepsilon_{-}\rangle =0. \end{array} \end{array}$$

Here, **0** donates a column zero vector. In the same way, we can infer

(21) $$\begin{array}{cc} \; & a_{1}|\varepsilon_{00}\rangle +a_{2}|\varepsilon_{10}\rangle -|\varepsilon_{+}\rangle =0,\\ \; & b_{1}|\varepsilon_{10}\rangle +b_{2}|\varepsilon_{11}\rangle -|\varepsilon_{+}\rangle =0,\\ \; & a_{1}|\varepsilon_{00}\rangle -a_{2}|\varepsilon_{10}\rangle -|\varepsilon_{-}\rangle =0,\\ \; & b_{1}|\varepsilon_{10}\rangle -b_{2}|\varepsilon_{11}\rangle +|\varepsilon_{-}\rangle =0,\\ \; & a_{1}|\varepsilon_{00}\rangle -|\varepsilon_{0}\rangle =0,\\ \; & b_{1}|\varepsilon_{01}\rangle =0,\\ \; & b_{2}|\varepsilon_{11}\rangle -|\varepsilon_{1}\rangle =0,\\ \; & a_{2}|\varepsilon_{10}\rangle =0. \end{array}$$

Hence, we can deduce the following result from Equation (21): $a_{2}=b_{1}=0$, $a_{1}=b_{2}=1$ and $|\varepsilon_{00}\rangle =|\varepsilon_{11}\rangle =|\varepsilon_{0}\rangle =|\varepsilon_{1}\rangle$. Substituting these results for the symbols in the Equations (17) and (18), we can obtain

(22) $$\begin{array}{cc} \; & U_{E}|0\rangle |\varepsilon \rangle =\left|0\rangle \right|\varepsilon_{00}\rangle ,\\ \; & U_{E}|1\rangle |\varepsilon \rangle =\left|1\rangle \right|\varepsilon_{00}\rangle . \end{array}$$

Consequently, Eve cannot distinguish {|0〉,|1〉} without introducing errors. If there is an error, it will be detected in the eavesdropping check. Then the protocol will be restarted without information disclosure, which makes Eve launch this kind of attack while acquiring nothing.

(**3**) **The intercept-resend attack**

Since Eve does not know the positions of the decoy photons, in order to obtain information from Bob, Eve intercepts $\bar{S_{C}}$ that Bob sends to Charlie. Eve then substitutes all the particles with fake ones randomly generated in {|0〉,|1〉,|+〉,|−〉} and sends them to Charlie. Suppose that the initial decoy particle state is |0〉, if Eve generates the particle randomly in the {|0〉,|1〉} basis, the probability that Eve’s attack will be detected is $\frac{1}{2}$, and if Eve generates the particle randomly in the {|+〉,|−〉} basis, the probability that Eve’s attack will be detected is $\frac{1}{2}$. In conclusion, the probability that Eve’s attack can be detected is $\frac{1}{2}\times \frac{1}{2}+\frac{1}{2}\times \frac{1}{2}=\frac{1}{2}$. When we use *n* decoy particles for eavesdropping, the probability of Eve’s attack being detected is 1 − ${\left(\frac{1}{2}\right)}^{n}$, which will approach 1 if *n* is large enough.

(**4**) **The measurement-resend attack**

Since Eve does not know the positions of the decoy photons, in order to obtain information from Bob, Eve intercepts $\bar{S_{C}}$ that Bob sends to Charlie. Subsequently, Eve randomly selects the Z basis or X basis to measure the intercepted particles and prepares new quantum states to send to Charlie based on the results of the measurements. Suppose that the initial decoy particle state is |0〉. If Eve chooses to measure with Z basis, Eve’s attack will incur no error, and if Eve chooses to measure with X basis, the probability that Eve’s attack will be detected is $\frac{1}{2}$. In conclusion, the probability that Eve’s attack can be detected is $\frac{1}{2}\times \frac{1}{2}=\frac{1}{4}$. When we use *n* decoy particles for eavesdropping, the probability of Eve’s attack being detected is 1 − ${\left(\frac{3}{4}\right)}^{n}$, which will approach 1 if *n* is large enough.

#### 2.3.2. Participant Attack

We now focus on the participant attack, a more severe threat to the protocol’s security. Naturally, in a quantum summation protocol with *n* participants, when any *n* − 1 participants conspire together, they can easily learn the left one’s secret input. Here, *n* is a positive integer equal or greater than 3. Therefore, we only analyze the participant attack from one dishonest participant.

In order to prevent their secret inputs from being known by others, each participant encrypts the *t*-th particle by using secret keys. He privately performs the Hadamard gate operation on the *t*-th particle. It is worth noting that if the protocol is information-theoretically secure, then for every input ρ, the output $\rho_{c}$ is a totally mixed state [38]. So, we determine whether the proposed protocol is information-theoretically secure by comparing the input density matrix with the output density matrix. The input state is related to the output state as follows:

(23) $$\rho_{out}=\sum_{i}p_{i}U_{i}\rho_{in}U_{i}^{†}$$

where $\rho_{in}$ is the density matrix of all possible initial input states, and $U_{i}$ is the corresponding unitary operator applied to the input state. We only analyze the relationship between the initial state and the output state of the *t*-th particle sent by Bob to Charlie in that Alice, Bob and Charlie play the same role in our protocol. Firstly, since the initial state of the *t*-th particle is at either |0〉 or |+〉, we can obtain

(24) $$\begin{array}{c} \rho_{in}=(\frac{1}{2}|0\rangle \langle 0|+\frac{1}{2}|+\rangle \langle +|) \end{array}$$

Then, after Alice and Bob performing the corresponding operators, the output density matrix should be in

(25) $$\begin{array}{c} \begin{array}{cc} \rho_{out} & =\frac{1}{4}\{H^{0}U^{0}[\frac{1}{2}U^{0}(\frac{1}{2}|0\rangle \langle 0|+\frac{1}{2}|+\rangle \langle +|)+\frac{1}{2}U^{1}(\frac{1}{2}|0\rangle \langle 0|+\frac{1}{2}|+\rangle \langle +|)]\}\\ \; & +\frac{1}{4}\{H^{1}U^{0}[\frac{1}{2}U^{0}(\frac{1}{2}|0\rangle \langle 0|+\frac{1}{2}|+\rangle \langle +|)+\frac{1}{2}U^{1}(\frac{1}{2}|0\rangle \langle 0|+\frac{1}{2}|+\rangle \langle +|)]\}\\ \; & +\frac{1}{4}\{H^{0}U^{1}[\frac{1}{2}U^{0}(\frac{1}{2}|0\rangle \langle 0|+\frac{1}{2}|+\rangle \langle +|)+\frac{1}{2}U^{1}(\frac{1}{2}|0\rangle \langle 0|+\frac{1}{2}|+\rangle \langle +|)]\}\\ \; & +\frac{1}{4}\{H^{1}U^{1}[\frac{1}{2}U^{0}(\frac{1}{2}|0\rangle \langle 0|+\frac{1}{2}|+\rangle \langle +|)+\frac{1}{2}U^{1}(\frac{1}{2}|0\rangle \langle 0|+\frac{1}{2}|+\rangle \langle +|)]\}\\ \; & =\frac{1}{4}(\frac{1}{4}|0\rangle \langle 0|+\frac{1}{4}|+\rangle \langle +|+\frac{1}{4}\left|1\rangle \langle 1\right|+\frac{1}{4}|-\rangle \langle -|)\\ \; & +\frac{1}{4}(\frac{1}{4}|+\rangle \langle +|+\frac{1}{4}\left|0\rangle \langle 0\right|+\frac{1}{4}|-\rangle \langle -|+\frac{1}{4}|1\rangle \langle 1|)\\ \; & +\frac{1}{4}(\frac{1}{4}|1\rangle \langle 1|+\frac{1}{4}|-\rangle \langle -|+\frac{1}{4}\left|0\rangle \langle 0\right|+\frac{1}{4}|+\rangle \langle +|)\\ \; & +\frac{1}{4}(\frac{1}{4}|-\rangle \langle -|+\frac{1}{4}\left|1\rangle \langle 1\right|+\frac{1}{4}|+\rangle \langle +|+\frac{1}{4}|0\rangle \langle 0|)\\ \; & =\frac{1}{4}(|0\rangle \langle 0|+|1\rangle \langle 1|+|+\rangle \langle +|+|-\rangle \langle -|)\\ \; & =\frac{1}{4}\left[\left(\begin{array}{cc} 1 & 0\\ 0 & 0 \end{array}\right)+\left(\begin{array}{cc} 0 & 0\\ 0 & 1 \end{array}\right)+\frac{1}{2}\left(\begin{array}{cc} 1 & 1\\ 1 & 1 \end{array}\right)+\frac{1}{2}\left(\begin{array}{cc} 1 & -1\\ -1 & 1 \end{array}\right)\right]\\ \; & =\frac{1}{2}\left(\begin{array}{cc} 1 & 0\\ 0 & 1 \end{array}\right)\\ \; & =\frac{1}{2}I \end{array} \end{array}$$

By Equation (25), we can see that the output of the *t* th particle after Bob performs quantum operators is just a totally mixed state. Namely, anyone, including the next participant, cannot acquire any information about Bob’s secret input $x_{B}$.

We then consider the case where the attack comes from TP. Since TP is a assumed a semi-honest third party in our protocol, he may try his best to learn the participants’ secret inputs without conspiring with anyone. Unlike an outside eavesdropper, TP can use various quantum resources to launch attacks, such as the Trojan horse attack, the intercept–resend attack and so on; besides, he can try to learn participants’ information from intermediate recorded by himself in the procedure of the protocol. We will explain that TP cannot learn any participants’ secret inputs. Similar to the density matrix analysis above, TP cannot learn the secret input of any participant from the recorded information. In addition, if TP wanted to intercept the transmitted particles without being detected, that would be impossible. Because, as analyzed in the outside attack above, every particle transmission requires a security check, any interception is detected in the security check.

Therefore, the proposed three-party quantum summation protocol is information-theoretically secure.

## 3. The Multi-Party Quantum Summation Protocol

### 3.1. Proposed Protocol

In this section, we will describe in detail how to generalize the three-party protocol to the multi-party protocol. We assume that there are *n* participants labeled $P_{1}$, $P_{2}$, …, $P_{n}$ (*n* > 2), and every participant $P_{i}$ (*i* = 1, …, *n*) has secret input $x_{i}$ = ($x_{i1}$, $x_{i2}$, …, $x_{im}$), $x_{it}$ϵ$\left\{0,1\right\}$, *t* = 1, 2, …, *m*. All participants want to obtain the summations $\sum_{i=1}^{n}x_{i}$ = ($x_{11}$⊕ …⊕$x_{n1}$, …, $x_{1m}$⊕ …⊕$x_{nm}$) and without revealing their secret information. Similarly, it should satisfy the requirements described in Section 2.1: correctness, fairness, security and privacy.

All participants still agree with the encoding forms described above:

(26) $$\begin{array}{cc} \; & {|0\rangle }_{0}={|0\rangle ,|0\rangle }_{1}=|+\rangle \\ \; & {|1\rangle }_{0}={|1\rangle ,|1\rangle }_{1}=|-\rangle \end{array}$$

with ${|0\rangle }_{0}=|0\rangle$ and ${|1\rangle }_{0}=|1\rangle$ denoting the classical bits 0 and 1 respectively.

**Step 1:** TP sends a secret key sequence $l_{i}$ to $P_{i}$ via the quantum key distribution protocol [11] in advance, where $l_{i}=\left(l_{i1},l_{i2},\ldots ,l_{im}\right),l_{it}\epsilon \left\{0,1\right\}$, *t* = 1, 2, …, *m*.

**Step 2:** TP encodes the secret key sequence $l_{1}$ previously shared with $P_{1}$ according to the above agreement, and he can obtain m copies of single photon states

$$S_{1}=\left\{{|0\rangle }_{l_{11}}{,|0\rangle }_{l_{12}},...,{|0\rangle }_{l_{1m}}\right\}.$$

TP randomly inserts m decoy photons randomly in $\left\{|0\rangle ,|1\rangle ,|+\rangle ,|-\rangle \right\}$ into sequence $S_{1}$. Define the new sequence as $\bar{S_{1}}$ Finally, TP sends $\bar{S_{1}}$ to $P_{1}$.

**Step 3:** After confirming that $P_{1}$ received all the particles $\bar{S_{1}}$, TP and $P_{1}$ check the transmissions between them for eavesdroppers. Concretely, TP first announces the insertion positions and the bases of the decoy photons to $P_{1}$. Then, based on the announced information, $P_{1}$ uses the correct basis to measure these decoy photons and publishes the measurement results to TP. Subsequently, according to their measuring results, TP checks whether eavesdroppers exist in the quantum channels. If the error rate is higher than the threshold determined by the channel noise, TP cancels this protocol and restarts it. Otherwise, TP proceeds to the next step.

**Step 4:** By extracting all the decoy photons from $\bar{S_{1}}$ and discarding them, $P_{1}$ can acquire the sequence $S_{1}$. Then, $P_{1}$ performs the unitary operation $U^{x_{1t}}$ on the *t*-th particle of $S_{1}$ according to his secret input $x_{1}$. When $P_{1}$ completes the encoding operation, the quantum states in $S_{1}$ would change to $\left\{|x_{11}\rangle_{l_{11}},|x_{12}\rangle_{l_{12}},\ldots ,{|x_{1m}\rangle }_{l_{1m}}\right\}$. Define the changed sequence as $S_{2}$. $P_{1}$ mixes $S_{2}$ with m decoy states randomly in $\left\{|0\rangle ,|1\rangle ,|+\rangle ,|-\rangle \right\}$ to form a new sequence $\bar{S_{2}}$. Finally, $P_{1}$ sends $\bar{S_{2}}$ to $P_{2}$.

**Step 5:** For *j* = 2, 3, …, *n*: when $P_{j}$ received the sequence $\bar{S_{j}}$ from $P_{j-1}$, $P_{j-1}$ checks the security of transmission with $P_{j}$, which similar to Step 3. After determining that the channel is secure, $P_{j}$ removes the decoy states and encodes his secret input $X_{j}$ similar to Step 4. Furthermore, $P_{j}$ encodes the information according to the secret key sequence $l_{j}$ sent by TP. To be clear, $P_{j}$ performs the unitary operation $H^{l_{jt}}$ on the *t* th particle of $S_{j}$. Then, $P_{j}$ mixes sequence $S_{j}$ and decoy photons randomly to form a new sequence $\bar{S_{j}}$, and sends it to $P_{j+1}$. Of note, the last participant $P_{n}$ sends the particle sequence $\bar{S_{n+1}}$ to TP.

**Step 6:** When TP has received the sequence $\bar{S_{n+1}}$, TP checks the security of transmission channel with $P_{n}$. TP obtains $S_{n+1}$ by extracts and discards decoy photons from $\bar{S_{n+1}}$. Then, TP computing $L_{1}$ = $\sum_{i=1}^{n}l_{i1}$, $L_{2}$ = $\sum_{i=1}^{n}l_{i2}$, …, $L_{m}$ = $\sum_{i=1}^{n}l_{im}$. If the result $L_{t}$ is 1, TP will perform the Hadamard operation on the t th particle of $S_{n}$. Otherwise, the particles in $S_{n}$ will not change. After the above steps, TP measures the particles with Z basis. Then, TP can obtain the summation $\sum_{i=1}^{n}x_{i1}$, $\sum_{i=1}^{n}x_{i2}$, …, $\sum_{i=1}^{n}x_{im}$. Finally, TP announces the summation result to $P_{1}$, $P_{2}$, …, $P_{n}$.

### 3.2. Correctness

It is correct for a secure multi-party summation protocol, which means that all participants can obtain the sum without revealing any secrets. In the following, we will show that the result of this protocol is the sum of their secret inputs.

Before the protocol is executed, the participants $P_{i}$, *i* = 1, …, *n* negotiate a coding rule whereby |0〉 and |1〉 represent the classical bits 0 and 1, respectively. Suppose that the initial state of the *t* th particle is ${|S\rangle }_{t}\epsilon \left\{|0\rangle ,|+\rangle \right\}$, *t* = 1, 2, …, *m*. In Steps 4 and 5, the encoding operation $H^{l_{it}}U_{Y}^{x_{it}}$ has been performed *n* − 1 times, that is, $H^{l_{2t}}U_{Y}^{x_{2t}},H^{l_{3t}}U_{Y}^{x_{3t}},\ldots ,H^{l_{nt}}U_{Y}^{x_{nt}}$, where $l_{it},x_{it}\varepsilon \left\{0,1\right\}$. After these encoding operations, by Equations (7)–(13), we can obtain the following:

(27) $$\begin{array}{c} \begin{array}{cc} |S^{*}\rangle_{1} & =H^{l_{n1}}U_{Y}^{x_{n1}}\ldots H^{l_{21}}U_{Y}^{x_{21}}U_{Y}^{x_{11}}{|S\rangle }_{1}\\ \; & =|x_{11}⊕x_{21}⊕\ldots \ldots x_{n1}\rangle_{l_{11}⊕l_{21}⊕\ldots ⊕l_{n1}}\\ |S^{*}\rangle_{2} & =H^{l_{n2}}U_{Y}^{x_{n2}}\ldots H^{l_{22}}U_{Y}^{x_{22}}U_{Y}^{x_{12}}{|S\rangle }_{2}\\ \; & =|x_{12}⊕x_{22}⊕\ldots \ldots x_{n2}\rangle_{l_{12}⊕l_{22}⊕\ldots ⊕l_{n2}}\\ \; & \ldots \\ |S^{*}\rangle_{m} & =H^{l_{nm}}U_{Y}^{x_{nm}}\ldots H^{l_{2m}}U_{Y}^{x_{2m}}U_{Y}^{x_{1m}}{|S\rangle }_{m}\\ \; & =|x_{1m}⊕x_{2m}⊕\ldots \ldots x_{nm}\rangle_{l_{1m}⊕l_{2m}⊕\ldots ⊕l_{nm}} \end{array} \end{array}$$

In Step 6, TP performs unitary operations on $|S^{*}\rangle_{t}$ based on the sum of secret keys. So, quantum states will change to the following form:

(28) $$\begin{array}{c} \begin{array}{cc} H^{L_{1}}{|S^{*}\rangle }_{1} & =H^{L_{1}}H^{l_{n1}}U_{Y}^{x_{n1}}\ldots H^{l_{21}}U_{Y}^{x_{21}}U_{Y}^{x_{11}}{|S\rangle }_{1}\\ \; & =|x_{11}⊕x_{21}⊕\ldots ⊕x_{n1}\rangle_{L_{1}⊕l_{11}⊕l_{21}⊕\ldots ⊕l_{n1}}\\ H^{L_{2}}{|S^{*}\rangle }_{2} & =H^{L_{2}}H^{l_{n2}}U_{Y}^{x_{n2}}\ldots H^{l_{22}}U_{Y}^{x_{22}}U_{Y}^{x_{12}}{|S\rangle }_{2}\\ \; & =|x_{12}⊕x_{22}⊕\ldots ⊕x_{n2}\rangle_{L_{2}⊕l_{12}⊕l_{22}⊕\ldots ⊕l_{n2}}\\ \; & \ldots \\ H^{L_{m}}{|S^{*}\rangle }_{m} & =H^{L_{m}}H^{l_{nm}}U_{Y}^{x_{nm}}\ldots H^{l_{2m}}U_{Y}^{x_{2m}}U_{Y}^{x_{1m}}{|S\rangle }_{m}\\ \; & =|x_{1m}⊕x_{2m}⊕\ldots ⊕x_{nm}\rangle_{L_{m}⊕l_{1m}⊕l_{2m}⊕\ldots ⊕l_{nm}} \end{array} \end{array}$$

Obviously, $L_{1}⊕l_{11}⊕l_{21}⊕\ldots ⊕l_{n1}=0,\ldots ,L_{m}⊕l_{1m}⊕l_{2m}⊕\ldots ⊕l_{nm}=0$. So, we can further obtain

(29) $$\begin{array}{c} \begin{array}{cc} \; & H^{L_{1}}|S^{*}\rangle_{1}={|x_{11}⊕x_{21}⊕\ldots ⊕x_{n1}\rangle }_{0}\\ \; & H^{L_{2}}|S^{*}\rangle_{2}={|x_{12}⊕x_{22}⊕\ldots ⊕x_{n2}\rangle }_{0}\\ \; & \ldots \\ \; & H^{L_{m}}|S^{*}\rangle_{m}={|x_{1m}⊕x_{2m}⊕\ldots ⊕x_{nm}\rangle }_{0} \end{array} \end{array}$$

Finally, TP performs the Z-basis measurement on the particles in the sequence $S_{n+1}$, and the particles collapse into classical information:

(30) $$x_{11}⊕x_{21}⊕\ldots ⊕x_{n1},x_{12}⊕x_{22}⊕\ldots ⊕x_{n2},\ldots ,x_{1m}⊕x_{2m}⊕\ldots ⊕x_{nm}.$$

Therefore, the correct result can be acquired by performing the protocol.

### 3.3. Security Analyse

For security, we use the same method to prevent outside and participant attacks in both three-party and multi-party quantum summation because the idea of the proposed two protocols is the same. We analyze the security of our multi-party protocol. Firstly, we prove that our protocol is resistant to outside attacks. Secondly, we show that participant attacks are ineffective for our protocol.

#### 3.3.1. Outside Attack

We analyze the possibility of an outside eavesdropper, Eve, obtaining the secret inputs from all participants.

Eve is considered to be able to launch various attacks using different quantum resources but not conspire. Next, we will explain that her attacks are ineffective. In order to obtain something useful information about participants’ secret inputs, Eve can utilize the particle transmission in steps 2, 4, and 5 to launch active attacks, such as the intercept–resend attack, the entanglement–measure attack, the measurement–resend attack and so on. However, we use decoy photons, which are randomly chosen from the two conjugate bases, Z basis and X basis, to detect the presence of an outside eavesdropper. This idea is derived from the unconditional security BB84 protocol [41]. It has been proven to be unconditionally secure [42]. We take the measurement-resend attack as an example here: if Eve tries to intercept the particles sent from $P_{i}$ to $P_{i+1}$ and measures them, then prepares fake quantum states based on the results to resend to $P_{\left(i+1\right)}$, he will introduce an extra error rate that would allow him to be detected during security checking. For a decoy photon chosen for detection, Alice reflects this particle back to TP with a probability of 1/2. Thus, Eve has a (1/2)*(1/2) = (1/4) probability of being detected. When we use *n* decoy particles for eavesdropping, the probability of Eve being caught turns into 1 − ${\left(3/4\right)}^{n}$, which will approach 1 if *n* is large enough. Therefore, if Eve launches active attacks during the particle transmissions, she will inevitably leave traces on the decoy photons and be detected by the eavesdropping check process since the locations and measurement basis of the decoy photons are not known until they are announced. In addition, since the transmission of particles in our protocol in the quantum channel is unidirectional, it is naturally resistant to the Trojan horse attacks.

#### 3.3.2. Participant Attack

In this subsection, we will sufficiently analyze two scenarios of participant attack: the participant attack from one or more dishonest parties and the participant attack from TP.

**Case 1:**  **The participant attack from one or more dishonest parties**

In the following, we will analyze two situations: one participant wants to learn the secret numbers from others; the other is more than one participant colluding together to learn secret numbers from others.

(**a**) **The participant attack from one dishonest party**

Without loss of generality, we assume that $P_{2}$ is the dishonest participant.

In Step 4, $P_{2}$ receives $S_{2}=\left\{|x_{11}\rangle_{l_{11}},|x_{1}2\rangle_{l_{12}},\ldots ,{|x_{1m}\rangle }_{l_{1m}}\right\}$ from $P_{1}$, but he cannot learn $P_{1}$’s secret message $X_{1}$ from $\bar{S_{2}}$ because $P_{2}$ does not know $l_{1}$ and he cannot conspire with TP, who knows the parties’ keys. In the protocol of this paper, the secret key represents a change in the measurement base, and the attacker does not know the key, so naturally, he will not know the corresponding measurement base. In addition, if $P_{2}$ tries to intercept particles transmitted between the remaining participants, he will be detected as an outside attacker because he does not know the position of the decoy photon and the measurement base.

Therefore, $P_{2}$ cannot obtain the secret input of $P_{1}$.

(**b**) **The participant attack from more than one dishonest party.**

If *n* − 1 participants collude, they can easily deduce the secret input of the other participant from the final summation result. Thus, the proposed multi-party quantum summation protocol can resist the collusion attack from at most *n* − 2 participants. Without loss of generality, we assume n−2 parties $P_{1},P_{2},\ldots ,P_{i-1},P_{i+1},\ldots ,P_{n-1}$ collude together to learn the secret input $x_{i}$ of $P_{i}$. In Step 5, $P_{i+1}$ can obtain $S_{i+1}={\{|x_{11}⊕\ldots ⊕x_{i1}\rangle }_{l_{11}⊕\ldots ⊕l_{i1}},\ldots ,|x_{1m}⊕\ldots ⊕x_{im}\rangle_{l_{1m}⊕\ldots ⊕l_{im}}\}$ from $P_{i}$.

By Equation (25), we can obtain the density matrix of the *t*-th particle output states in the sequence $S_{i+1}$:

(31) $$\rho_{out}=\sum_{i}p_{i}U_{i}\rho_{in}U_{i}^{†}=\frac{1}{2}I$$

Since the output of the *t*-th particle after $P_{i}$ performs the corresponding operators is just a totally mixed state, no one can obtain any information about $P_{i}$’s secret input, even if $P_{1},P_{2},$$\cdots ,P_{i-1},P_{i+1},\cdots ,P_{n-1}$ collude together to deduce $x_{i}$. Furthermore, from a measurement perspective, since $P_{1},P_{2},\cdots ,P_{i-1},$$P_{i+1},\cdots ,P_{n-1}$ do not know the key, $P_{i+1}$ cannot know the measurement basis corresponding to the quantum sequence and thus cannot obtain the secret information $x_{i}$ of $P_{i}$. In Step 6, $P_{1},P_{2},\cdots ,P_{i-1},P_{i+1},$$\cdots ,P_{n-1}$ can learn the summation result from TP. They can only obtain the value of $x_{i}⊕x_{n}$ and cannot deduce $x_{i}$ because they have no knowledge about the secret input $x_{n}$.

Therefore, $P_{1},P_{2},\ldots ,P_{i-1},P_{i+1},\ldots ,P_{n-1}$ cannot learn the secret information of $P_{i}$, even if they conspire.

**Case 2:**  **The participant attack from TP**

Let us now consider TP attacks. In our protocol, TP is assumed as a semi-honest third party, which means that TP can perform all sorts of attacks by using various quantum resources, and attempt learn the secret inputs of participants from the information he records while the protocol is in progress but non-collusive. Next we will show that TP cannot obtain secret inputs of any participants without being detected.

In Step 6, TP can obtain $S_{n+1}={\{|x_{11}⊕\cdots ⊕x_{n1}\rangle }_{l_{11}⊕\cdots ⊕l_{n1}},\cdots ,|x_{1m}⊕\cdots$$⊕x_{nm}\rangle_{l_{1m}⊕\cdots ⊕l_{nm}}\}$. TP obtains the sum of all secret inputs after encoding and measuring the quantum sequence $S_{n+1}$. Although TP knows the keys of all participants, he is still unable to learn any secret inputs from them. After encoding, the quantum states sequence $S_{n+1}$ will become ${\{|x_{11}⊕\cdots ⊕x_{n1}\rangle }_{0},|x_{12}⊕\cdots ⊕x_{n2}\rangle_{0},\cdots ,|x_{1m}⊕\cdots ⊕x_{nm}\rangle_{0}\}$, and the effect of the key is eliminated. Thus, without colluding with the participants, TP does not derive clues about secret information from the sum. Even if TP measured the particles directly after obtaining the sequence $S_{n+1}$, he would still not acquire the desired result. It is worth noting that TP can infer the measurement base corresponding to each particle in the sequence $S_{n+1}$ from the secret keys of all participants. Because it is still a mixture of information from all participants, TP does not separate the secret inputs of any participants from $S_{n+1}$. If TP attempts to intercept transited particles between any two participants, he will be detected as an outside attacker. Furthermore, even if TP does intercept the transmitted particles, it will not be able to obtain the desired secret information because it did not know the location of the decoy photons.

Therefore, TP cannot obtain any participants’ secret inputs without being caught.

## 4. Comparisons

In this section, we compare the previous quantum summation protocols with our multi-party quantum summation from the quantum resource, the quantum operations, particle transmission mode, quantum measurements and the qubit efficiency in Table 2.

The qubit efficiency of secure quantum communication was introduced by Cabello [43], defined as

(32) $$\eta =\frac{c}{q+b},$$

where *c* is the total number of the classical plaintext message bits, *q* represents the number of the used qubits and *b* denotes the number of classical bits exchanged for decoding the message. For simplicity, we suppose that the number of participants is *N*, the length of the summation is *m*, and *m* decoy particles are employed to check eavesdrop.

Ref. [32] presented a secure multi-party summation protocol based on single photons in both polarization and spatial-mode degrees of freedom. In their protocol, $\frac{m}{2}$ single photons are used in both polarization and spatial-mode degrees of freedom for encoding, mN decoy particles are used for detecting the presence of eavesdroppers, and finally, TP announces the result will cost *m* classical bits. So, the qubit efficiency is $\eta =\frac{2}{2N+3}$.

Ref. [35] proposed a multi-party summation protocol within a *d*-level quantum system. In their protocol, $P_{1}$ generates 2(m+δ)*d*-level single photons, of which (m+δ) photons are used to check whether the communication is secure, and δ photons are used to check the security of communication with $P_{2},P_{3},\ldots ,P_{N}$. For ease of calculation, we assume that, ideally, δ = *m*. $P_{1}$ restores all photons in his hand to the original orders will cost 2(N−1)m classical bits, and announcing the result when the summation is complete will cost *m* classical bits. So, the qubit efficiency is $\eta =\frac{1}{2N+3}$.

**Table 2:** Comparison between previous quantum summation protocols and ours.

	Ref. [32]	Ref. [35]	Ref. [34]	Ref. [27]	Ref. [44]	Ref. [28]	Ref. [30]	Ref. [31]	Our Protocol
Quantum resource	single photons in both polarization and spatial-mode degree of freedom	d-level single-photon state	d-level single-photon state	three-particle entangled state	d-level N-particle entangled state	N-particle entangled state	Bell state	phase GHZ state	single-photon state
Quantum operations	Single-photon operators	Unitary operations	Two unitary operations ($X_{d}$ and $Y_{d}$)	CNOT and Hadamard operators	Quantum Fourier transformand and Pauli operators	Pauli and Hadamard operators	NOT and identity operators	No	Pauli and Hadamard operators
Particle transmission mode	circle-type	circle-type	circle-type	tree-type	tree-type	tree-type	circle-type	star-type	circle-type
Quantum measurements for TP	Single-photon projective measurements	single qudit measurements	Single qudit measurement	No	No	Single-photon projective measurements	Single-photon projective measurements	No	Single-photon projective measurements
Quantum measurement for participants	No	No	No	Single-photon projective measurements	Single qudit measurements	No	Single-photon projective measurements	Computational basis measurements	No
Qubit efficiency	$\frac{2}{2N+3}$	$\frac{1}{2N+3}$	$\frac{1}{2N+3}$	$\frac{1}{4N-1}$	$\frac{1}{3N-2}$	$\frac{1}{3N-2}$	$\frac{1}{5N}$	$\frac{1}{4N^{4}+3N}$	$\frac{1}{2N+2}$

Ref. [34] employed single particles to construct a multi-party summation protocol in a *d*-level quantum system, where *d* is restricted to odd primes. Since the summation length of their protocol is 1, we assume for convenience of calculation that the number of decoy particles is also 1 for each participant. In their protocol, (N+1) decoy particles are used for checking the presence of eavesdroppers, $P_{1},\ldots ,P_{N}$ announces that their encrypted number to TP for summation will cost *N* classical bits, and TP announces the result will cost 1 classical bit. So, the qubit efficiency is $\eta =\frac{1}{2N+3}$.

Ref. [27] utilized three-party entangle states to construct a modulo-2 summation protocol. In their protocol, there are mN particles for key generation, mN particles for checking the honesty of the initiator, m(N−1) decoy particles for eavesdropping detection, and participants need to announce mN classical bits to get the summation. So, the qubit efficiency is $\eta =\frac{1}{4N-1}$.

Ref. [44] proposed a secure multi-party summation protocol by employing *d*-level *n*-particle entangle states. $P_{1}$ prepares mN particles for encoding and (N−1)m decoy particles for eavesdropping detecting. In addition, $P_{2},\cdots ,P_{N}$ need to announce $P_{1}$ their measurements to acquire the sum, and this process will cost (N−1)m particles. So, the qubit efficiency is $\eta =\frac{1}{3N-2}$.

Ref. [28] used N-particle entangled states to construct a secure multi-party summation protocol. There are two cases for their protocol since the difference between the two cases is only in the number of particles prepared, and the difference is not significant. We will only consider the *n* − 1 mod 2 = 0 case. In their protocol, mN particles for encoding and (N−1)m decoy particles are employed to detect eavesdropping during the transmission of the particle sequence $S_{i}$ from $P_{1}$ to $P_{i}$, where *i* = 2, …, *N*. Moreover, (N−1)m decoy particles are needed to detect eavesdropping during the particle sequence $S_{i}^{\prime }$ sent back to $P_{1}$ by $P_{i}$. So, the qubit efficiency is $\eta =\frac{1}{3N-1}$.

In the protocol of Ref. [30], *d*-level single quantum systems are employed to design a multi-party quantum summation. Within their protocol, there are 2mN particles for encoding and 2mN decoy particles for eavesdropping detection. Furthermore, participants need to announce mN classical bits to compute the summation. So, the qubit efficiency is $\eta =\frac{1}{5N}$.

Ref. [31] proposed a multi-party modulo summation protocol based on GHZ states. For ease of calculation, we assume that each group of *N* copies, except the final group, where each copy consists of *N* qubits. In their protocol, $(4N^{3}+1)N$ particles for verifiable generation, *N* bits for summation, participants announce their result will cost *N* bites. So, the qubit efficiency is $\eta =\frac{1}{4N^{4}+3N}$.

In our protocol, there are mN particles for previous keys distribution, *m* particles for encoding, and mN particles for eavesdropping detection. In addition, when the sum is obtained, TP needs to announce *m* classical bits. Then, the qubit efficiency of the presented protocols is $\eta =\frac{1}{2N+2}$. In our work, only single photons, unitary operation, and single-particle measurement are introduced.

According to Table 2, we can conclude the following: ➀ As for the qubit efficiency, our protocol is second only to the protocol in Ref. [32]. However, Ref. [32] has a drawback in terms of security. The secret input encoded on single photons is not encrypted, and a malicious participant $P_{i}$ (*i* = 1, …, *n* − 1) can obtain $P_{i+1}$’s secret by an intercept-resend attack. To solve this problem, Ref. [33] proposed a modification. At the beginning of the protocol, each participant first shares a set of keys with TP by using some secure quantum key distribution protocols, and then encodes the sum of secrets and keys on the photons instead. Although this modification increases safety, it makes Ref. [32] no longer advantageous in terms of qubit efficiency compared to our protocol. Our protocol can guarantee safety and convenience with excellent qubit efficiency. ➁ As for the quantum resource, this protocol outperforms protocols in Refs. [27,28,30,31,32,44], as the preparations of multiple-particle entangled states and single photons in both polarization and spatial-mode degree of freedom are more difficult than those of single photon states. In addition, in Refs. [28,30], every participant needs quantum generators to generate quantum states, which will make it more difficult to implement the protocol. ➂ As for quantum operations, with the current technology, it is difficult to achieve the manipulation in the high-dimensional quantum system as in Refs. [26,27,34,35,44]. Our protocol is carried out in two-dimensional Hilbert space, which is feasible with current technology [45]. ➃ As for quantum measurements, this protocol exceeds protocols in Refs. [27,30,34,35,44], since the single qudit measurements in Refs. [34,35,44] are much more complicated to realize than the single-photon projective measurements, and in Refs. [27,30,44], every participant requires quantum measurement devices in multiple different bases, which makes them more cumbersome to implement than our protocol. So, our protocol is more efficient and feasible compared to other protocols.

## 5. Conclusions

In summary, we presented a novel and efficient protocol for secure multi-party quantum summation. In our approach, *n* participants complete this task with the help of a semi-honest TP. TP is responsible for preparing and distributing single-photon states and performing quantum measurements, while participants only employ unitary operations to encode their secret data and transfer the particle to the next participant. The proposed protocol can also resist various attacks, such as the entanglement–measure attack, the measurement–resend attack, and the denial-of-service attack. Furthermore, considering the practical security and technical feasibility, our protocol takes single-photon states as quantum resources and only needs simple single-particle operators and single-photon measurements. Therefore, the proposed protocol is feasible with the current technology and of high efficiency.

## Figures and Tables

**Table 1:** Encoding operations on the sequence.

$x_{1}$ = (0, 1, 0)	$x_{2}$ = (0, 1, 0)	$x_{3}$ = (0, 1, 1)
${|0\rangle }_{0}\overset{U_{x_{A1}}}{⟶}{|0\rangle }_{0}$	$\overset{U_{l_{B1}}U_{x_{B1}}}{\to }{|0\rangle }_{0}$	$\overset{U_{l_{C1}}U_{x_{C1}}}{\to }{|0\rangle }_{0}$
${|0\rangle }_{1}\overset{U_{x_{A2}}}{⟶}{|1\rangle }_{0}$	$\overset{U_{l_{B2}}U_{x_{B2}}}{\to }{|0\rangle }_{1}$	$\overset{U_{l_{C2}}U_{x_{C2}}}{\to }{|1\rangle }_{0}$
${|0\rangle }_{0}\overset{U_{x_{A3}}}{⟶}{|1\rangle }_{1}$	$\overset{U_{l_{B3}}U_{x_{B3}}}{\to }{|1\rangle }_{1}$	$\overset{U_{l_{C3}}U_{x_{C3}}}{\to }{|0\rangle }_{0}$

## Data Availability

My manuscript has no associated data.
